# Evolution of extreme stomach pH in bilateria inferred from gastric alkalization mechanisms in basal deuterostomes

**DOI:** 10.1038/srep10421

**Published:** 2015-06-08

**Authors:** Meike Stumpp, Marian Y. Hu, Yung-Che Tseng, Ying-Jeh Guh, Yi-Chih Chen, Jr-Kai Yu, Yi-Hsien Su, Pung-Pung Hwang

**Affiliations:** 1 Institute of Cellular and Organismic Biology, Academia Sinica, Taipei, Taiwan R.O.C.; 2 Helmholtz Centre for Ocean Research Kiel (GEOMAR), Kiel, Germany; 3 Institute of Physiology, Christian Albrechts University Kiel, Kiel, Germany; 4 Department of Life Sciences, National Taiwan Normal University, Taipei, Taiwan R.O.C

## Abstract

The stomachs of most vertebrates operate at an acidic pH of 2 generated by the gastric H^+^/K^+^-ATPase located in parietal cells. The acidic pH in stomachs of vertebrates is believed to aid digestion and to protect against environmental pathogens. Little attention has been placed on whether acidic gastric pH regulation is a vertebrate character or a deuterostome ancestral trait. Here, we report alkaline conditions up to pH 10.5 in the larval digestive systems of ambulacraria (echinoderm + hemichordate), the closest relative of the chordate. Microelectrode measurements in combination with specific inhibitors for acid-base transporters and ion pumps demonstrated that the gastric alkalization machinery in sea urchin larvae is mainly based on direct H^+^ secretion from the stomach lumen and involves a conserved set of ion pumps and transporters. Hemichordate larvae additionally utilized HCO_3_^−^ transport pathways to generate even more alkaline digestive conditions. Molecular analyses in combination with acidification experiments supported these findings and identified genes coding for ion pumps energizing gastric alkalization. Given that insect larval guts were also reported to be alkaline, our discovery raises the hypothesis that the bilaterian ancestor utilized alkaline digestive system while the vertebrate lineage has evolved a strategy to strongly acidify their stomachs.

The highly acidic pH of most vertebrate stomachs is believed to protect against environmental pathogens and the mechanisms of gastric acidification are well described^1^,^2^,^3^. The H^+^/K^+^-ATPase (HKA) in luminal membranes of parietal cells drives H^+^ ions into the stomach lumen leading to an accumulation of hydrochloric acid^4^. This enables vertebrates to generate highly acidic conditions in their digestive systems. In contrast, midgut alkalization in lepidopteran and dipteran insect larvae, is energized by an V-H^+^-ATPase (VHA) coupled to processes that result in net export of H^+^ from the luminal space^5^,^6^,^7^. These observations demonstrate that different gastric pH regulatory mechanisms have evolved in the animal kingdom. An interest is thus raised in regard to whether the highly acidic or alkaline digestive system is an ancestral character of the bilaterian.

Although marine invertebrates were described to have slightly acidic to neutral pH in their digestive systems^8^,^9^ virtually nothing is known regarding gastric pH regulation of their larval stages. In a previous study we demonstrated that larvae of the green sea urchin, *Strongylocentrotus droebachiensis*, have alkaline conditions in their digestive systems^10^. This study also demonstrated that their digestive enzymes are adapted to alkaline conditions and the digestive efficiency is reduced when gastric pH is decreased. Gastric pH homeostasis is a critical process in order to maintain optimum digestion efficiencies to support nutrition of the developing larvae. Although sea urchins are prime organisms to study regulatory mechanisms controlling early development^11^, many functions of the larval gut remain unexplored.

## Results

### Pharmacological characterization of the gastric pH regulatory systems in sea urchin and acorn worm larvae

**Figure 1:**
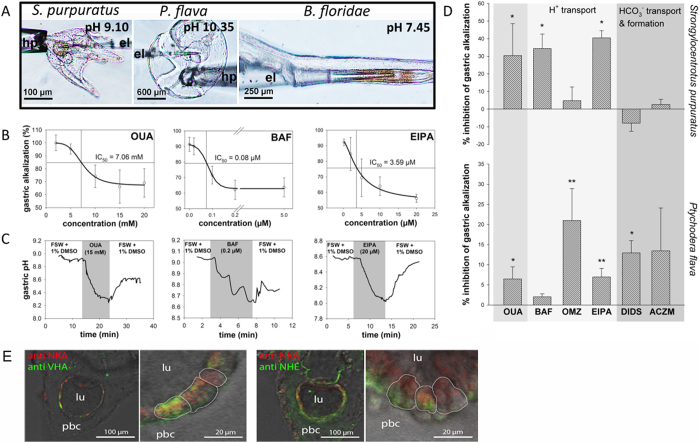
Characterization of the gastric pH regulatory machinery in ambulacrarian larvae. (**A**) Microelectrode (el) pH measurements in larval digestive systems of the three species indicating highly alkaline digestive systems pluteus (7-15 dpf) and tornaria (10 -20 dpf) larvae and less alkaline conditions in *B. floridae* larvae (feeding stage 3 dpf). (**B**) Dose response curves for the inhibition of gastric alkalization in pluteus larvae were determined for the inhibitors ouabain (OUA), bafilomycin (BAF) and ethyl-isopropyl amiloride (EIPA) with respective IC_50_ values. (**C**) Real time traces of gastric pH during application of inhibitors and 5 mM Na^+^ solutions (Supplemental material Figure S1) and washout. (**D**) Effects of inhibitors including OUA, BAF, EIPA, omeprazole (OMZ), 4,4“-diisothiocyanatostilbene-2,2′-disulphonic acid (DIDS) and acetazolamide (ACZM) as well as 0 mM HCO_3_^−^, 5 mM Na^+^ and 0 mM K^+^ seawater solutions (for raw values including control experiments see Supplemental material Figure S1; n = 5-7) on the gastric alkalization machinery. (**E**) Immunocytochemical analyses in sea urchin pluteus larvae demonstrate the sub cellular localization of Na^+^/K^+^-ATPase (NKA), V-type H^+^-ATPase (VHA) and Na^+^/H^+^-exchanger (NHE3) immunoreactivity in the stomach epithelium of *Strongylocentrotus purpuratus* plutei. Dotted lines indicate the position of stomach cells. Values are presented as mean ± SE and asterisks denote significant differences (* p < 0.05, ** p < 0.001). holding pipette: hp; lumen:lu; primary body cavity: pbc; stomach: st; oesophagus: oes; intestine: int.

Using H^+^ selective microelectrodes we determined gastric pH at the larval stages of the sea urchin *Strongylocentrotus purpuratus*, the hemichordate *Ptychodera flava* and the cephalochordate *Branchiostoma floridae* (Fig. 1A). In contrast to acidic digestive systems found in most vertebrates, the gastric pH of echinoid pluteus and hemichordate tornaria larvae was highly alkaline and the pH was 9.10 ± 0.02 (n = 11) and 10.35 ± 0.01 (n = 5) in *S. purpuratus* and *P. flava*, respectively. However, the gastric pH of the cephalochordate *B. floridae* was 7.45 ± 0.09 (n = 7), slightly more acidic compared to the environmental pH of approximately 8 (Fig. 1A). The alkaline pH of the sea urchin and hemichordate larval guts was progressively established during development as the guts became functional in the pluteus and tornaria larvae (Supplemental material Figure S1 A + B). We next explored the gastric pH regulatory mechanisms in sea urchin larvae using specific inhibitors for acid-base relevant ion transporters. Our results showed that gastric alkalization in sea urchin larvae is inhibited by ouabain and bafilomycin, specific inhibitors for the Na^+^/K^+^-ATPase (NKA) and the V-type H^+^-ATPase (VHA), respectively (Fig. 1B+C). In addition, gastric alkalization was decreased by treatments with ethyl-isopropyl amiloride (EIPA, a specific inhibitor of Na^+^-dependent H^+^ exchangers (NHEs)) (Fig. 1B+C) as well as artificial seawater with reduced Na^+^ (5 mM) and K^+^ (0 mM) concentrations (Supplemental material Figure S1 C+D), indicating the involvement of NHEs as well as NKA in this process. No effect on gastric alkalization was found using inhibitors against enzymes involved in HCO_3_^−^ transport and formation (4.4´-diisothiocyanatostilbene-2,2´-disulphonic acid (DIDS) and Acetazolamide) as well as a gastric H^+^/K^+^-ATPases (HKA) inhibitor omeprazol^12^ (Fig. 1D). The inhibition of gastric alkalization by inhibitors happened within few minutes (Fig. 1C) corroborating with our previous study that demonstrated high permeability of the pluteus ectoderm^13^. In accordance to findings in another sea urchin species (*S. droebachiensis*)^14^, high concentrations of NKA were found in luminal membranes of the stomach epithelium of *S. purpuratus* larvae (Fig. 1E), indicating that this epithelium is capable of active ion transport^14^. Antibodies designed against the molluscan H^+^-ATPase and teleost NHE3 indicated positive VHA immunoreactivity in luminal membranes and positive NHE3 immunoreactivity in epithelial membranes facing the primary body cavity (PBC) (Fig. 1E and Supplemental material Figure S2).

Similar to the situation in echinoid pluteus larvae, the gastric alkalization machinery of hemichordate tornaria larvae depended on NKA and EIPA sensitive cationic exchange mechanisms to remove protons from the stomach lumen. However, tornaria larvae additionally used a HCO_3_^−^ transport mechanism, which was sensitive to DIDS, to maintain alkaline gastric pH (Fig. 1D and Supplemental material Figure S1 E). Contrasting to pluteus larvae, gastric alkalization in tornaria larvae was insensitive to bafilomycin, but instead to omeprazole, a specific inhibitor for the gastric HKA.

### Identification of ion pumps and transporters energizing gastric alkalization

**Figure 2:**
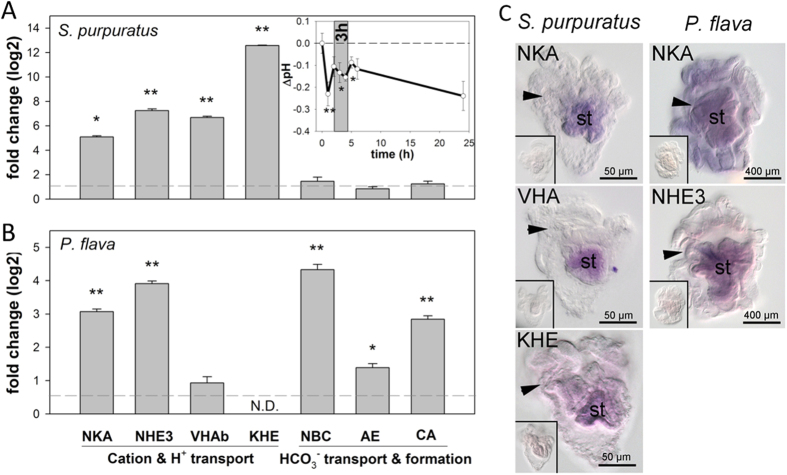
Identification of acid-base transporters in *Strongylocentrotus purpuratus* and *Ptychodera flava* using acidification experiments. Relative change in transcript abundance of putative acid-base transporters determined by qPCR in *S. purpuratus* (**A**) and *P. flava* (**B**) larvae exposed to control (pH 8.1) and acidified conditions (pH7). Time series over 24 h demonstrating relative changes in gastric pH in response to acidified seawater (pH 7) in sea urchin larvae (**A**; insert). The grey area indicates the sampling time point (3 h) for gene expression studies. Values are presented as mean ± SE and asterisks denote significant differences (* p < 0.05, ** p < 0.001). (**C**) *In situ* hybridization of selected acid-base transporters including Na^+^/K^+^-ATPase (NKA), V-type H^+^-ATPase (VHA), K^+^/H^+^-exchanger (KHE) and Na^+^/H^+^-exchanger 3 (NHE3) expressed in the digestive system of *S. purpuratus* and *P. flava* larvae raised under control conditions (insert: negative control using sense probe). Arrows indicate the location of the larval mouth. Stomach: st.

**Figure 3:**
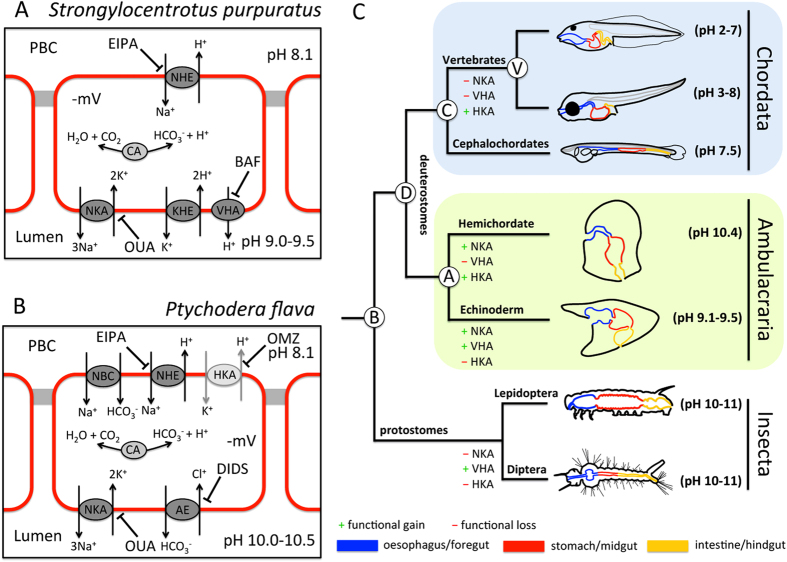
A) Hypothetical models of gastric alkalization in echinoid pluteus larvae (**A**) and hemichordate tornaria larvae (**B**). Gastric alkalization in *S. purpuratus* larvae is cation based to achieve net export of protons from the stomach lumen whereas *P. flava* larvae additionally employ a luminal import of HCO_3_^−^. Gastric alkalization is energized by NKA and VHA in sea urchin and NKA and a putative HKA (light grey) in hemichordate larvae. Bars indicate the inhibition of ion pumps and transporters by specific inhibitors. **C**) Phylogenetic tree depicting the different sections of the digestive systems in duterostome larval stages and a dipteran larva. *S. purpuratus* and *Ptychodera flava* representing the ambulacraria have alkaline conditions (pH 9-10) in their stomachs. Neutral to acidic conditions are a character of chordate larvae including the cephalochordate *B. floridae*, teleost fish^37^ and amphibians^38^. Alkaline digestive systems were also found in the anterior midgut of some insect larvae. The differential importance of the three major ion pumps including NKA, VHA and HKA responsible for gastric pH regulation are highlighted for the different groups. A: Ambulacraria ancestor; B: bilateralia ancestor; C: Chordate ancestor; Deuterostome ancestor; V: Vertebrate ancestor.

We next used qPCR to investigate expression changes of genes encoding acid-base relevant transporters in pluteus and tornaria larvae (Supplemental material Figure S3) upon challenge of the gastric pH regulatory machinery with acidified seawater conditions (insert in Fig. 2). Seawater acidification experiments were used to increase the H^+^ gradient between stomach lumen and the extracellular space. If larvae try to stabilize stomach pH under acidified conditions this will stimulate the gastric pH regulatory machinery indicated by an up-regulation of acid-base regulatory genes. Sea urchin larvae compensated for a drop in stomach pH within few hours (Fig. 2A). This compensation reaction was accompanied by increased transcript abundance of several ion transport genes, including NKA, VHA, NHE3 and a KHE that are normally expressed in stomach epithelial cells (Fig. 2A+C). No response was observed for genes encoding transporters involved in HCO_3_^−^ transport. These findings correlate with our inhibitor studies and suggest a cation-based alkalization mechanism in the digestive system of echinopluteus larvae (summarized in Fig. 3A).

Inhibitor and gene expression studies further demonstrate that in addition to cation-based luminal alkalization mechanisms, tornaria larvae of the hemichordate *P. flava* utilize anion (e.g. HCO_3_^−^ and Cl^−^) exchange pathways in order to create a highly alkaline digestive environment (Figs. 1D and 2B,C). Gene expression studies also suggest an involvement of carbonic anhydrase (CA) and Na^+^-dependent HCO_3_^−^ co-transport in this alkalization process (summarized in Fig. 3B).

## Discussion

Alkaline digestive conditions may represent an evolutionary advantage for marine herbivorous larvae to facilitate breakdown of plant and algal proteins under alkaline conditions^15^. Moreover, alkaline conditions >pH 9.5 in larval digestive systems can be regarded as an efficient defense mechanism against environmental pathogens because most marine protists and viruses are killed by alkaline conditions exceeding pH 9.5^16^. Furthermore, our measurements provide compelling evidence that sea urchin and acorn worm larvae have evolved sophisticated pH regulatory mechanisms to control gastric pH homeostasis. On the opposite side these unique digestive features were proposed to represent a key feature explaining sensitivity towards climate change induced reductions in seawater pH by impairing digestion and exerting an additional energetic costs to the larvae in future oceans^14^. The different mechanisms to generate alkaline digestive systems in sea urchin and hemichordate larvae show many parallels to differential midgut alkalization mechanisms found in Lepidoptera and Diptera insect larvae. Our findings for the sea urchin pluteus larvae suggest a primarily cation-based alkalization mechanism in the digestive system of echinopluteus larvae (summarized in Fig. 3A). A similar cationic-based ion regulatory machinery has been proposed for midgut alkalinization in Lepidopteran insect larvae, where an apical V-type H^+^-ATPase energizes secondary active transport processes including K^+^/H^+^antiport and subsequent export of protons across the basolateral membrane^5^,^18^. In contrast to the Lepidopteran alkalization system which is insensitive to ouabain, pluteus larvae additionally use an NKA-dependent pathway to energize gastric alkalization. Interestingly, NKA is localized in basolateral membranes of most epithelia with only two exceptions where NKA is localized in luminal/apical membranes, namely the mosquito anterior midgut^19^ and the retinal pigment epithelium^20^. We suggest that NKA localized in luminal membranes of the sea urchin larval stomach creates the electrochemical gradient energizing the export of H^+^ across membranes facing the PBC in exchange for Na^+^ *via* NHEs. NHE-based proton secretion mechanisms are an ancient pathway of pH and ionic regulation^21^ which are believed to be energetically favorable in marine environments due to high external [Na^+^] (≈460 mM) compared to low intracellular [Na^+^] (≈30 mM)^22^.

Although proton secretion mechanisms as found in the sea urchin larva are also an important pathway in *P. flava*, these hemichordate larvae additionally utilize HCO_3_^−^ transport mechanisms to support strong alkalization of their stomachs (midgut). Boudko *et al.*^6^ proposed a similar anion-based model for strong luminal alkalization of the mosquito (Diptera) anterior midgut where HCO_3_^−^ is accumulated in the luminal space in exchange for Cl^−^. Also in this insect example the V-type H^+^-ATPase located in basolateral membranes is thought to energize luminal alkalization. Interestingly, gastric alkalization in tornaria larvae is not sensitive to bafilomycin, but to the HKA inhibitor omeprazole^23^, suggesting that HKA plays a critical role in gastric alkalization in hemichordate larvae. The gastric HKA is well described in vertebrates, and is the major player in gastric acid secretion^24^. Vertebrate NKAs and HKAs share a high degree of similarity (Supplemental material Figure S4), and gastric HKA may even function as NKA under certain conditions^25^. In fact, a single amino acid (human lysine_791_) is postulated to define the affinity of the enzyme for H^+^ transport instead of Na^+^ 26. Phylogenetic analyses based on α subunit sequence similarities suggested that the diversity of all vertebrate αNKAs and αHKAs evolved from a single ancient deuterostome αNKA by gene and genome duplications^27^.

Similar to the situation in vertebrate systems gastric pH homeostasis in sea urchin and hemichordate larvae is controlled by specialized stomach epithelial cells. While the vertebrate digestive system is composed of at least 11 different cell types^28^, the stomach epithelium of the sea urchin larvae have been reported to consist of only two cell types^29^. Thus, the relatively simple organization of the pluteus larval digestive system together with this first set of functional markers provided here could help to identify regulatory pathways underlying the differentiation of the digestive system in early deuterostomes. Although potential gene candidates for gastric alkalization were identified in the present work, a true loss of function approach using gene knock down would be an important future task to clarify the contribution of specific transporters to gastric pH regulation. Furthermore, our results raise the hypothesis that the alkaline digestive system may be present in the common ancestor of bilateria. During evolution leading to the chordate lineage, the ancestral chordate employed acidic digestive system and the acidity became extreme in the vertebrate lineage (Fig. 3C). Additional investigations in a wider range of duterostome and protostome larvae, such as lophotrochozoans, are needed to provide more definite conclusions whether alkaline digestive systems represent an ancestral trait of the bilaterian larva. This information will provide an important basis to better understand the evolution of gastric pH regulatory systems and will help to disentangle regulatory processes leading to the functional formation of digestive systems in animals.

## Methods

### Larval cultures - *Strongylocentrotus purpuratus*

Adult *Strongylocentrotus purpuratus* were collected from the Californian coast (Kerckoff marine Laboratory, California Institute of Technology), transferred to the Institute of Cellular and Organismic Biology and maintained in re-circulating natural seawater system at 14 °C and in the dark with regular water changes twice to three times per week. Animals were fed with *Laminaria* sp. Spawning in *Strongylocentrotus purpuratus* was induced by shaking. One male and one female were used for each culture and cultures were continuously started once to twice a week. The general condition of the animals was continuously monitored and only larvae from healthy cultures (determined by morphology, means of larval movement, cilia activity and esophageal muscle contractions) were used. Larval cultures (2 L glass beakers) had initial concentrations of 15 Larvae mL^−1^ and were continuously aerated using small motorized paddles. Larvae were kept in a temperature controlled incubator at 14 °C, a salinity of 33 and with a light-dark cycle of 12 h/12 h. From three days post-fertilization (dpf) on, larvae were daily fed with *Rhodomonas* spp. at concentrations ranging around 10,000 cells mL^−1^. Food concentrations were determined using a Fuchs-Rosenthal counting cell chamber. The water was changed three times per week. The inhibitor experiments were conducted on larvae between 20 to 40 dpf, where relatively stable alkaline gastric conditions were observed.

### Larval cultures - *Ptychodera flava*

Adult animals were collected at Penghu Island, Taiwan, and transferred to the ICOB at Academia Sinica, Taipei, Taiwan, where they were kept in a recirculating natural seawater system at 25 °C and a salinity of 33. Spawning in *Ptychodera flava* was induced by heat shocks at 35 °C for 15 min in combination with diluted, inactivated sperm water.

Tornaria larvae were kept at densities of 10 larvae mL^−1^ glass beakers at 25 °C, a salinity of 33 and a light-dark cycle of 12 h/12 h. Food (10,000 cells mL^−1^) was added daily after the water was exchanged. The inhibitor experiments were conducted on Heider stage larvae between 7 to 12 dpf, where relatively stable alkaline gastric conditions were observed.

### Selective ion electrode technique

Microelectrode construction and measurements of stomach pH were essentially conducted as described previously^10^,^13^. Briefly, the H^+^ selective microelectrodes with tip diameters of 3-5 μm were front loaded with a H^+^ ionophore (H^+^ ionophore III, Sigma Aldrich) and back filled with a KCL based electrolyte (300 mM KCL, 50 mM NaPO_4_, pH 7). To measure gastric pH, the larvae were placed into a perfusion chamber mounted on an inverted microscope and were held in position using a holding pipette. The pH micoelectrode was gently inserted through the oesophagus using a micromanipulator.

### Inhibitor experiments – *S. purpuratus*/*P. flava*

Larvae were bathed in a range of inhibitors (Quabain, DIDS, EIPA, Acetazolamide, Omeprazol, Bafilomycin A) and artificial seawater (ASW) test solutions (5 mM sodium, 0 mM bicarbonate, 0 mM potassium; ASW compositions are provided in Supplemental material Table S2) to determine the transporters participating in gastric alkalization. Inhibitors were dissolved in DMSO and subsequently diluted in 0.4 μm filtered seawater. Finally culture seawater including larvae were mixed with 2x concentrated, pH adjusted (pH 8.1) test solution in a relation of 1:1 to the final test concentration. Ten to twenty larvae were incubated in inhibitors in a volume of 1 mL for about 1.5 to 2 hours with a parallel control batch of larvae that were incubated in the appropriate DMSO concentrations only. The DMSO concentration never exceeded 1% and had no significant impact on gastric pH. Larvae bathed in artificial seawater with 5 mM sodium were incubated for only 15 minutes since the effect on the larvae was immediate and a longer incubation time resulted in larval death. Larvae bathed in 0 mM bicarbonate were incubated over night with no visible effect on larval morphology, movement or gut contractions. During incubations, the larvae were kept in a temperature constant water bath (14 °C for *S. purpuratus*, 25 °C for *P. flava*). Larvae were then placed onto the microscope chamber in the test solution and the gastric pH was quickly measured within 5 to 10 min per 5 to 8 larvae using pH sensitive microelectrodes. For inhibitor time traces, we used a temperature controlled perfusion bath as previously described. Inhibitor and test solutions with the chosen incubation times did not visibly impact larval morphology or behavior in terms of cilia-based swimming movement or occurrence of oesophagal muscle contractions. We expected the inhibitors to reach the gastric epithelium from the luminal side through continuous swallowing of inhibitor containing seawater and the extracellular matrix (ECM) side through diffusion into the ECM through the epidermal epithelium since our inhibitors were in the range of 0.2 to 0.8 kDa and the epidermal epithelium was previously documented to be leaky for molecules up to a size of 4 kDa^13^.

### Acidification experiments – *S. purpuratus*

Acidifcation experiments were conducted twice. To determine the time course of gastric pH compensation, the first experiment (A) measured gastric pH along the period of 24 hours using 16 dpf larvae. The second experiment (B) was conducted for sampling of larvae for gene expression analysis over a timeframe of 3 hours using 17 dpf larvae from the same larval culture as in A. The pH for the low pH treatment was adjusted by injecting pure CO_2_ until the desired pH of 7.0 was reached (Supplemental material Table S1). Larvae from the cultures were directly transferred into gas tight glass bottles filled with seawater of the respective pH (control pH 8.1 or low pH 7.0). The pH inside the incubation bottles remained constant along the entire incubation period. A relatively low pH was used in these acute low pH exposure experiments to ensure a pH regulatory response that can be detected on the transcriptional level. The incubation time of up to 24 hours did not have any visible impact on the larval morphology or behavior. pH_NBS_ was either measured using microelectrodes (see SIET section for details) that were cross calibrated with a WTW-pH-meter equipped with a SenTix 81 pH electrode (exp A) or using the WTW pH-meter directly (exp B). The pH meter was calibrated using standard NBS buffers (pH 7.00 and pH 10.01). Total alkalinity (*A*_T_) was determined using the photometric method by Sarazin *et al.*^30^. Seawater carbonate chemistry for the experiments was calculated using *A*_T_ and pH_NBS_ with constants by Mehrbach *et al.*^31^ refitted by Dickson and Millero^32^.

#### Experiment A

*S. purpuratus* larvae were incubated at concentrations of 10 larvae mL^−1^ in 2 mL Eppendorf Tubes (without bubbles) which were submerged in the respective seawater pH in Falcon tubes (50 mL) in a water bath to ensure stable pH and temperature conditions throughout the experiment. For each time point one Eppendorf Tube of the respective treatments was used (2 treatments x 8 time points = 16 tubes). The pH_NBS_ did not change within the eppendorf tubes during the cause of the experiment. Gastric pH was determined in five larvae per eppendorf tube (control and low pH) at each time point (0, 1, 2, 3, 4, 5, 6 and 24 hours).

#### Experiment B

*S. purpuratus* and *P. flava* larvae were incubated at approximately 15 (*S. purpuratus*) and 30 (*P. flava*) larvae mL^−1^ in 60 mL Duran glass bottles that were completely filled with culture water (without bubbles) to ensure stable pH conditions throughout the experiments (Supplemental material Table S1). During incubations, Duran bottles were placed in an incubator at 14 °C for *S. purpuratus* and at 25 °C for *P. flava*, respectively. The experiment was conducted with five independent replicates per treatment per time point (e.g. 5 replicates x 2 treatments x 2 timepoints = 20 bottles). Larvae were sampled at 2 and 3 hours incubation from each treatment (control pH 8.1 and low pH 7.0). Larvae from each replicate bottle were quickly filtered and transferred in 1 mL culture water to 1.5 mL Eppendorf Tubes. Larvae were then hand-centrifuged for 30 s, excess seawater was discarded and larvae were immediately shock-frozen in liquid nitrogen. Each sample contained approximately 800 pluteus and 1000 tornaria larvae, and samples were stored at -80 °C for further analysis. pH_NBS_ was measured in each replicate bottle just before sampling and *A*_T_ samples were randomly collected from 6 samples per treatment.

### RNA preparation

Larvae were homogenized in lysis buffer using a Tissue lyser (Qiagen). RNA was extracted using the illustra triplePrep RNA extraction kit (GE Healthcare) DNA contamination was removed with DNase I (Promega, Madison, WI, USA). The mRNA for the RT-PCR was obtained with a QuickPrep Micro mRNA Purification Kit (Amersham Pharmacia, Piscataway, NJ, USA) according to the supplier protocol. The amount of mRNA was determined by spectrophotometry (ND-2000, NanoDrop Technol, Wilmington, DE), and the mRNA quality was checked by running electrophoresis in RNA gels. All mRNA pellets were stored at -80 °C.

### qRT-PCR

The mRNA expressions of target genes were measured by qPCR with the Roche LightCycler® 480 System (Roche Applied Science, Mannheim, Germany). Primers for all genes were designed (Supplemental material Table S3 and S4) using Primer Premier software (vers. 5.0; PREMIER Biosoft International, Palo Alto, CA). Hemichordate sequence information was obtained from the *P. flava* transcriptome ( http://molas.iis.sinica.edu.tw/hemichordate). PCRs contained 3.2 ng of cDNA, 50 nM of each primer, and the LightCycler® 480 SYBR Green I Master (Roche) in a final volume of 10 μl. All qPCRs were performed as follows: 1 cycle of 50 °C for 2 min and 95 °C for 10 min, followed by 40 cycles of 95 °C for 15 s and 60 °C for 1 min (the standard annealing temperature of all primers). PCR products were subjected to a melting-curve analysis, and representative samples were electrophoresed to verify that only a single product was present. Control reactions were conducted with sterile water to determine levels of background and genomic DNA contamination. The standard curve of each gene was confirmed to be in a linear range with UBQ1 and EF1a as reference gene. For the normalization factor (NF_n_) the geometric mean of these reference genes was calculated.

### *In situ* hybridization and Immuno-histological analyses

*In situ* hybridization and immunostaining were performed as previously described using larvae raised under control conditions^33^. The primary antibodies used were a monoclonal antibody IgGα5 raised against the avian α subunit of the Na^+^/K^+^-ATPase (Developmental Studies Hybridoma Bank, University of Iowa), a polyclonal antibody designed against synthetic peptides corresponding to the subunit A region (SYSKYTRALDEFYDK) of the molluscan V-type H^+^-ATPase^34^ and a polyclonal antibody raised against a 16-residue synthetic peptide (VAPSQRAQTRPPLTAG) of the teleost Na^+^/H^+^-exchanger 3 35. Immuno-fluorescence staining was examined with a Leica confocal microscope (SP5) and *in situ* hybridization signals were monitored with a Zeiss Imager A1.

For immunoblotting, 20 μL of crude extracts from 10,000-20,000 larvae were used. Proteins were fractionated by SDS-PAGE on 10% polyacrylamide gels, according to Lämmli^36^, and transferred to PVDF membranes (Millipore), using a tank blotting system (Bio-Rad). Blots were pre-incubated for 1 h at room temperature in TBS-Tween buffer (TBS-T, 50 mM Tris -HCl, pH 7.4, 0.9% (wt/vol) NaCl, 0.1% (vol/vol) Tween20) containing 5% (wt/vol) blocking reagent (Roche, Mannheim, Germany). Blots were incubated with the primary antibody (see previous section) diluted 1:250-500 at 4 °C overnight. After washing with TBS-T, blots were incubated for 2 h with horseradish conjugated goat anti-rabbit IgG antibody (diluted 1:1,000-2,000, at room temperature; Amersham Pharmacia Biotech). Protein signals were visualized by using the enhanced chemiluminescence system (ECL, Amersham Pharmacia Biotech) and recorded using Biospectrum 600 imaging system (UVP, Upland, CA, USA).

## Additional Information

**How to cite this article**: Stumpp, M. *et al.* Evolution of extreme pH in bilaterian digestive systems inferred from gastric alkalization mechanisms in basal deuterostomes. *Sci. Rep.* **5**, 10421; doi: 10.1038/srep10421 (2015).

## Supplementary Material

Supplementary Information
