# Supplementary material for: Evolution of extreme stomach pH in bilateria inferred from gastric alkalization mechanisms in basal deuterostomes
Source: Sci Rep. 2015 Jun 8;5:10421. doi: 10.1038/srep10421 (PMC4458843; doi:10.1038/srep10421)
Supplement: Supplementary Information [file srep10421-s1.doc]

**Supplementary Material to:**

**Evolution of extreme stomach pH in bilateria inferred from gastric alkalization mechanisms in basal deuterostomes**

Meike Stumpp, Marian Y. Hu, Yung-Che Tseng, Ying-Jeh Guh, Yi-Chih Chen, Jr-Kai Yu, Yi-Hsien Su and Pung-Pung Hwang

Table S1 Seawater physiochemical parameters during acidification experiments

| pH treatment | pH | *A*_T_  (mM) | *p*CO_2_ (µatm) | *C*_T_  (mM) | Temp °C | Sal |
| --- | --- | --- | --- | --- | --- | --- |
| *S. purpuratus* |  |  |  |  |  |  |
| 8.1 | 8.04 ± 0.01 | 2.02 ± 010 | 489 ± 16 | 1.89 ± 0.09 | 14 | 31 |
| 7.0 | 6.98 ± 0.02 | 2.06 ± 0.01 | 6456 ± 482 | 2.30 ± 0.10 | 14 | 31 |
| *P. flava* |  |  |  |  |  |  |
| 8.2 | 8.25 ± 0.002 | 2.21 ± 0.02 | 332 ± 4 | 1.91 ± 0.02 | 25 | 31 |
| 7.0 | 6.99 ± 0.015 | 2.20 ± 0.02 | 7683 ± 265 | 2.40 ± 0.01 | 25 | 31 |

Table S2 Artificial seawater solutions

| ion | 5 mM Na^+^ | 0 HCO_3_^-^ | 0 K^+^ |
| --- | --- | --- | --- |
| Na^+^ | 5 | 474 | 430 |
| K^+^ | 9.9 | 9.9 | 0 |
| Mg^2+^ | 53.3 | 53.3 | 53.3 |
| Ca^2+^ | 10.3 | 9.9 | 15.3 |
| Cl^-^ | 488.6 | 519.3 | 478.6 |
| SO_4_^2-^ | 28 | 28 | 28.2 |
| HCO_3_^-^ | 2.35 | 0 | 2.35 |
| NMDG^+^ | 443 |  |  |
| Hepes |  | 5 |  |
| pH | 8.2 | 8.2 | 8.2 |
| Osmolality | 1004 ± 10 | 1009 ± 10 | 1007 ± 10 |
| Artificial seawater (ASW) solutions (concentrations given in mmol Kg^-1^) | | | |

**Table S3 Sea urchin (*Strongylocentrotus purpuratus***) primer sequences used for qPCR

| **Gene name** | **Abbreviation** | **primer** | **Amplicon length** | **Accession number** |
| --- | --- | --- | --- | --- |
|  |  |  |  |  |
| Na^+^/K^+^-ATPase | NKA | [F] 5'-GTCTTCTTGGGTGTATCCTTCT-3' | 148 bp | XM_001202868.1 |
|  |  | [R] 5'-TAGCAGTCAAGGTCAGACATAC-3' |  |  |
| Na^+^/H^+^-exchanger 3 | NHE3 | [F] 5'-TGCTCACCCTGTCTAGTATTC-3' | 173 bp | XM_001178217.1 |
|  |  | [R] 5'-GCTGAGTGATGTAGTCCCTAA-3' |  |  |
| V-type H^+^-ATPase | VHA | [F] 5'-GAAAGATGGAAGCGGCTAAAG-3' | 119 bp | XM_001176838.1 |
|  |  | [R] 5'-ATTGTCCAGGATAACCAGAGG-3' |  |  |
| Na^+^/HCO_3_^-^ cotransporter | NBC | [F] 5'-GTAGAGTTCCATCCAGAGGTT-3' | 172 bp | NM_001079551.1 |
|  |  | [R] 5'-TTGTGGGTAGGTCCAGATTAG-3' |  |  |
| transmembrane and coiled-coil domains 3 (Tmco3) | KHE | [F] 5'-GTTGGTGGAGCCCATTAGAGA-3' | 168 bp | SPU_020239 |
|  |  | [R] 5'-CTCAGGGTTGTGACGCTTAGA-3' |  |  |
| Cl^-^/HCO_3_^-^ exchanger 2 | AE2 | [F] 5'-GTTCCTAACAGCAGAGAAGATG-3' | 176 bp | XM_788556.3 |
|  |  | [R] 5'-GTGAGAGACATGCAAAGTAGAG-3' |  |  |
| Carbonic anhydrase | CA | [F] 5'-GCGGTGTTATCCATCTTTCAC-3' | 163 bp | XM_003726241.1 |
|  |  | [R] 5'-GGGAAGCCACTCCTAATTACT-3' |  |  |
| Elongation factor 1A | EF1a | [F] 5'-GAGAGTTTGAGGCTGGTATCT-3' | 155 bp | NP_001116969 |
|  |  | [R] 5'-GACCTCCCTGACGATTTCTTT-3' |  |  |
| Ubiquitin-1 | UBQ1 | [F] 5'-GAGAGGAATCCTGAGGTATCG-3' | 160 bp | XM_777914.3 |
|  |  | [R] 5'-GTAGCGCATTATAACCACCTG-3' |  |  |

**Table S4 Acorn worm (*Ptychodera flava***) primer sequences used for qPCR

| **Gene name** | **Abbreviation** | **primer** | **Amplicon length** |
| --- | --- | --- | --- |
|  |  |  |  |
| Na^+^/K^+^-ATPase | NKA | [F] 5'-CTCACAGTGGGCAGTGTTAAAG-3' | 148 bp |
|  |  | [R] 5'-GTGCACCCTTCATGACCAATAG-3' |  |
| Na^+^/H^+^-exchanger 3 | NHE3 | [F] 5'-ATCGTCTTGGAAGCCGGATATT-3' | 161 bp |
|  |  | [R] 5'-TCCATTTCTACGTCCACCCATC-3' |  |
| V-type H^+^-ATPase | VHA | [F] 5'-GTACACTGACTTGGCAACCATC-3' | 149 bp |
|  |  | [R] 5'-TCTGACCTTCTGTGATGTACCC-3' |  |
| Na^+^/HCO_3_^-^ cotransporter | NBC | [F] 5'-GCCCTAGAGACGTTGGAAGTTA-3' | 148 bp |
|  |  | [R] 5'-AGTGATACGACGTTCCCTTCTG-3' |  |
| transmembrane and coiled-coil domains 3 (Tmco3) | KHE | [F] 5'-TAAGCTCCCAGTCACACAGTAA-3' | 170 bp |
|  |  | [R] 5'-AATCACCACGACAAAGGTCAAG-3' |  |
| Cl^-^/HCO_3_^-^ exchanger 2 | AE2 | [F] 5'-ACGAAGAATTCGATGGAGACTG-3' | 162 bp |
|  |  | [R] 5'-CACATTGCGTACATTGGTCTTG-3' |  |
| Carbonic anhydrase | CA | [F] 5'-GATGGTGGGAAGCTGATGAAAG-3' | 154 bp |
|  |  | [R] 5'-GTGTTCGTGAGTGTCATGGTAG-3' |  |
| Elongation factor 1A | EF1a | [F] 5'-GCTGATTGTGCTGTGTTGATTG-3' | 154 bp |
|  |  | [R] 5'-GCTCTGTGTTGTCCATCTTGTT-3' |  |
| Ubiquitin-1 | UBQ1 | [F] 5'-GCAGGAAATGATGAGAAGTCAGG-3' | 149 bp |
|  |  | [R] 5'-CTGAGAATGGGTTTGTACCAAGG-3' |  |

**
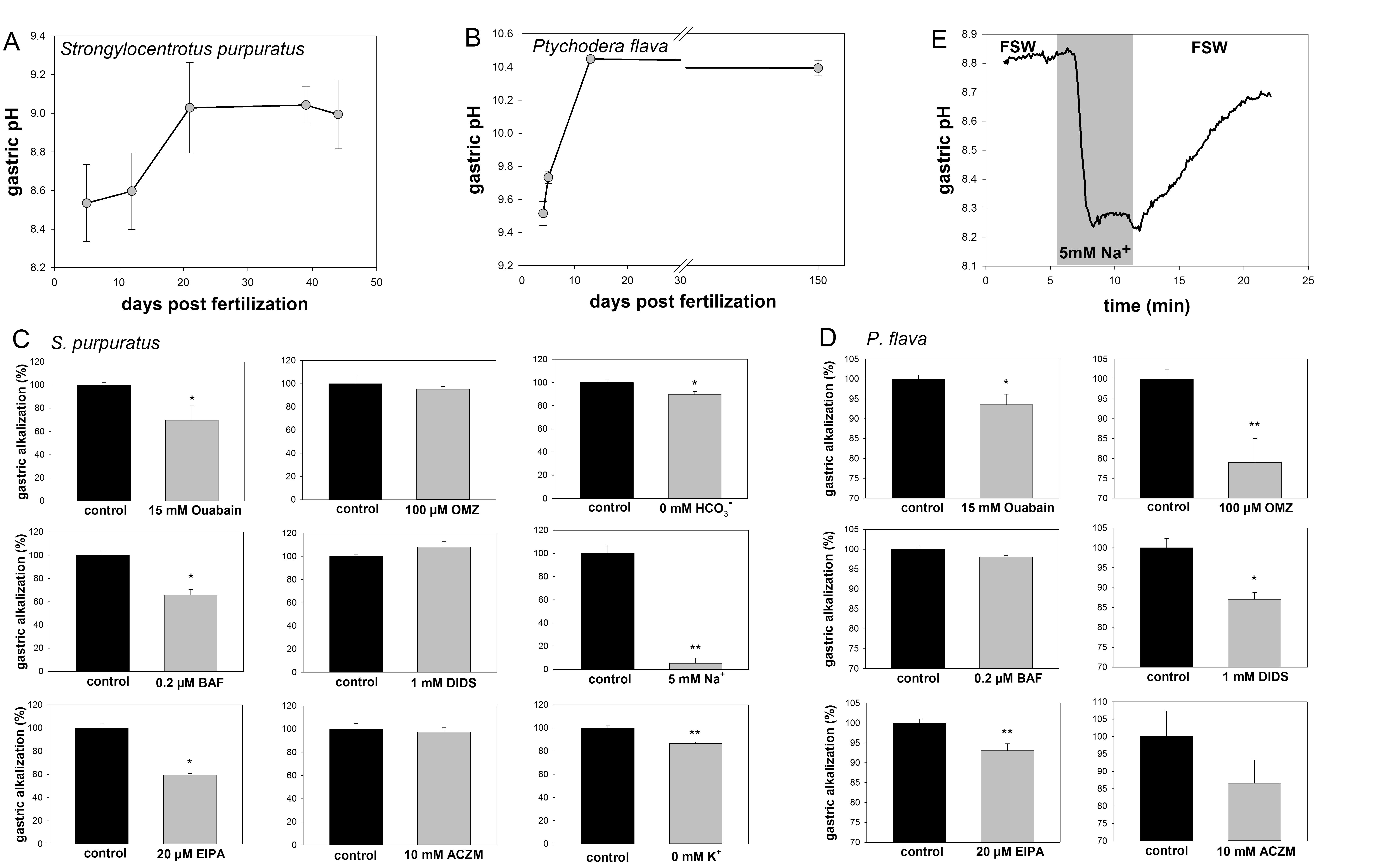
Figure S1** Gastric pH along larval development of *Strongylocentrotus purpuratus* (A) and *Ptychodera flava* (B). Values are presented as mean ± SE (n = 4; with 4 -6 animals from each of the four culture bottles). Raw data of the inhibitor experiments showing the control and the inhibitor groups from which the relative change in gastric inhibition (Fig. 1 D) was calculated for *S. purpuratus* (C) and *P. flava* (D). Values are presented as mean ± SE and asterisks denote significant differences (* p<0.05, ** p<0.001; n= 5-7). Real time trace of gastric pH in *S. purpuratus* larvae during exposure to 5 mM Na^+^ ASW (E). In response to artificial seawater containing 5 mM Na^+^ gastric pH drops rapidly by 0.6 pH units to seawater pH levels of 8.2. During washout with natural seawater the gastric pH recovers slightly below control conditions.


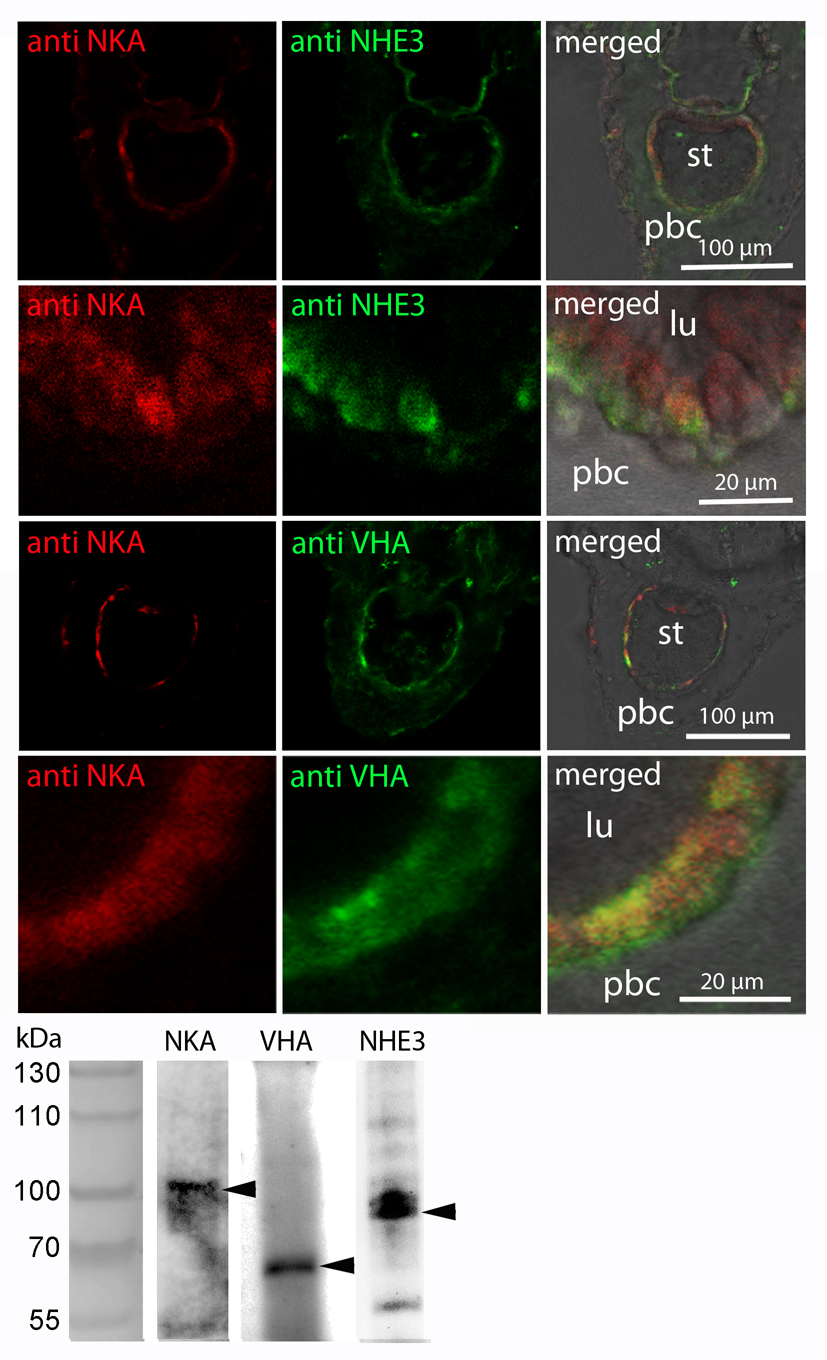


**Figure S2** Immunohistochemical analyses of acid-base transporters in sea urchin pluteus larvae. Colocalization of putative acid-base transporters in sea urchin larvae using antibodies designed against Na^+^/K^+^-ATPase (NKA) (chicken), V-Type-H^+^-ATPase (squid) and Na^+^/H^+^-exchanger 3 (fish) (upper panel). Flourescence images are presented separately (left and middle lane) and in a merged version including a bright field image (right lane). Western blot analysis demonstrates specific immunoreactivity with proteins in the predicted size range (lower panel).

**
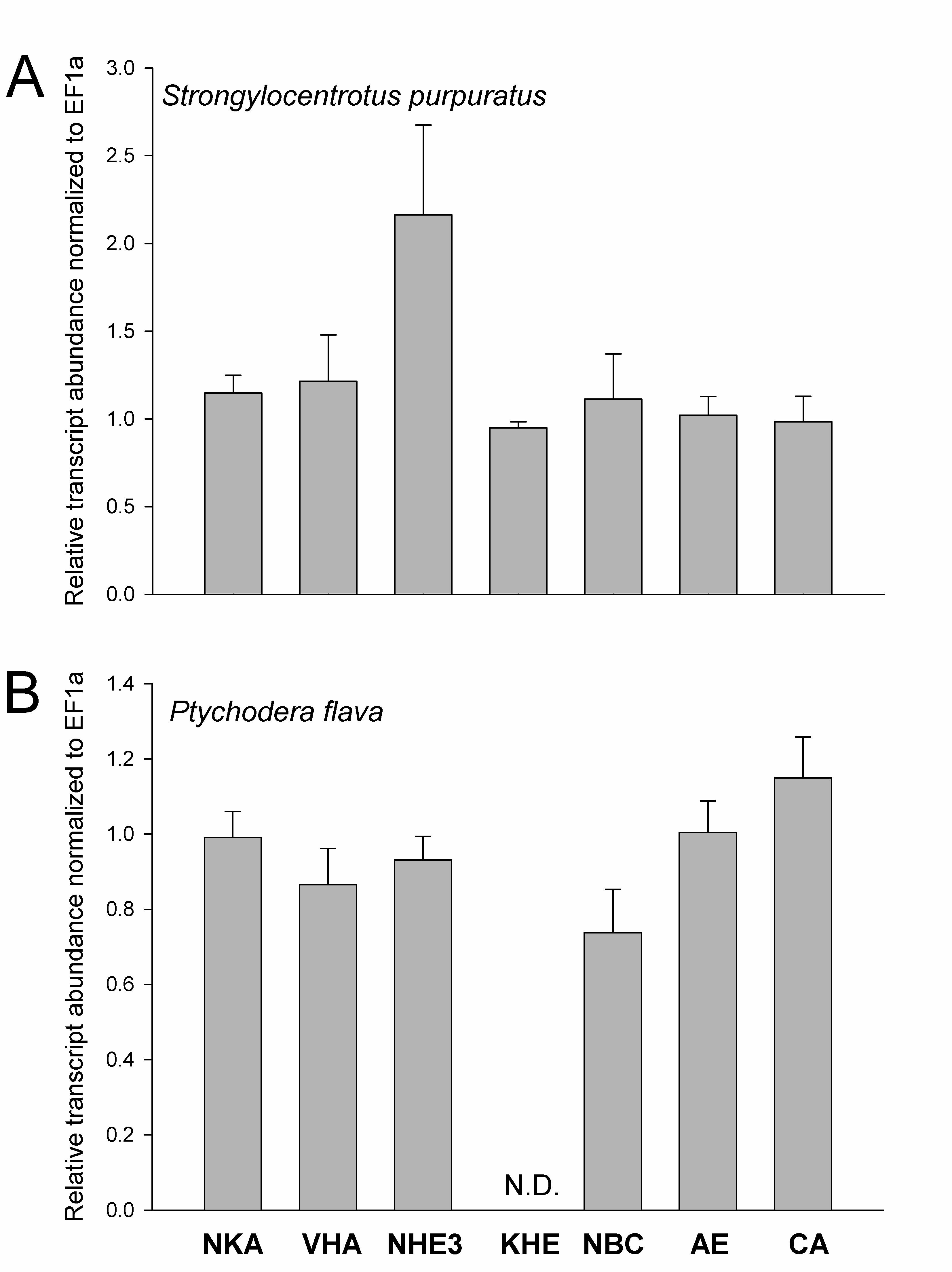
**

**Figure S3** Relative transcript abundance of acid-base regulatory genes under control conditions. Thranscript abundance of Na^+^/K^+^-ATPase (NKA), V-Type-H^+^-ATPase (VHA), Na^+^/H^+^-exchanger 3 (NHE3), K^+^/H^+^ exchanger (KHE), Na^+^/HCO_3_^-^ cotransporter (NBC), anion exchanger (AE) and carbonic anhydrase (CA) in sea urchin (*S. purpuratus*) pluteus larvae (A) and hemichordate (*P. flava*) tornaria larvae (B). Transcript concentrations of the putative KHE were not detectable in *P. flava* larvae. Transcripts were normalized to the housekeeping matrix of UBQ1 and EF1a and bars represent mean ± SE.

**
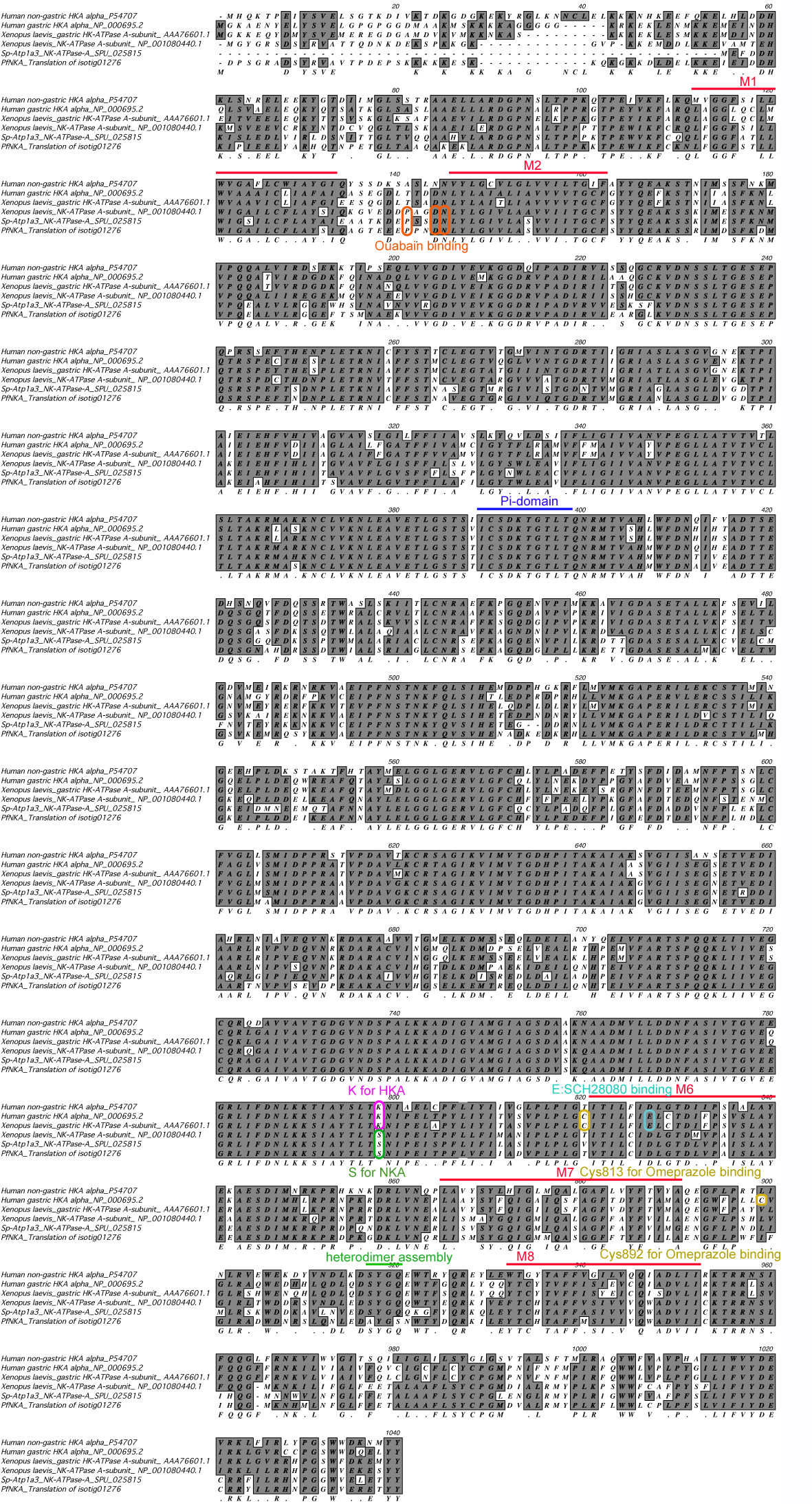
**

**Figure S4** Alignment of deduced Na^+^/K^+^-ATPase (NKA), gastric-type H^+^/K^+^-ATPase (g-HKA) and non-gastric H^+^/K^+^-ATPase (n-g-HKA) aminoacid sequences. Ouabain binding between transmembrane domain 1 and 2 (M1, M2) is highly specific for NKAs including those from sea urchin and hemichordate. Omeprazole binding to Cys_813_ and Cys_892_ is highly conserved among g-HKA, and suggest no effect on NKAs or n-g HKAs. Additionally, the binding site for Sch28080, another specific inhibitor for g-HKA is indicated in this alignemnt in tranasmembrane domain M6. The affinity for H^+^ or Na^+^ in KHAs and NKAs is determined by K (lysine) or S (serine) at position 798. Other functional domains including phosphorylation site (Pi) and heterodimer assembly site are highlighted.
